# Correlations Between Gut Microbiota Composition, Medical Nutrition Therapy, and Insulin Resistance in Pregnancy—A Narrative Review

**DOI:** 10.3390/ijms26031372

**Published:** 2025-02-06

**Authors:** Robert-Mihai Enache, Oana Alexandra Roşu, Monica Profir, Luciana Alexandra Pavelescu, Sanda Maria Creţoiu, Bogdan Severus Gaspar

**Affiliations:** 1 Department of Radiology and Medical Imaging, Fundeni Clinical Institute, 022328 Bucharest, Romania; robert-mihai.enache@rez.umfcd.ro; 2 Department of Morphological Sciences, Cell and Molecular Biology and Histology, Carol Davila University of Medicine and Pharmacy, 050474 Bucharest, Romania; oana-alexandra.rosu@rez.umfcd.ro (O.A.R.); monica.profir@rez.umfcd.ro (M.P.); luciana.pavelescu@umfcd.ro (L.A.P.); 3 Department of Oncology, Elias University Emergency Hospital, 011461 Bucharest, Romania; 4 Department of Surgery, Carol Davila University of Medicine and Pharmacy, 050474 Bucharest, Romania; bogdan.gaspar@umfcd.ro; 5 Surgery Clinic, Bucharest Emergency Clinical Hospital, 014461 Bucharest, Romania

**Keywords:** insulin resistance, gestational diabetes mellitus, microbiota, microbiome, dysbiosis, medical nutrition therapy

## Abstract

Many physiological changes accompany pregnancy, most of them involving metabolic perturbations. Alterations in microbiota composition occur both before and during pregnancy and have recently been correlated with an important role in the development of metabolic complications, such as insulin resistance and gestational diabetes mellitus (GDM). These changes may be influenced by physiological adaptations to pregnancy itself, as well as by dietary modifications during gestation. Medical nutritional therapy (MNT) applied to pregnant women at risk stands out as one of the most important factors in increasing the microbiota’s diversity at both the species and genus levels. In this review, we discuss the physiological changes during pregnancy and their impact on the composition of the intestinal microbiota, which may contribute to GDM. We also discuss findings from previous studies regarding the effectiveness of MNT in reducing insulin resistance. In the future, additional studies should aim to identify specific gut microbial profiles that serve as early indicators of insulin resistance during gestation. Early diagnosis, achievable through stool analysis or metabolite profiling, may facilitate the timely implementation of dietary or pharmaceutical modifications, thereby mitigating the development of insulin resistance and its associated sequelae.

## 1. Introduction

The hallmark of gestational diabetes mellitus (GDM) is glucose intolerance that appears or is discovered during pregnancy. It is linked to considerable hazards for maternal and fetal outcomes during pregnancy, birth, and beyond, and it affects 5–20% of expectant mothers globally with a prevalence continuously rising [1,2].

Gestational diabetes mellitus (GDM) significantly increases the risk of various maternal complications during pregnancy. These complications include birth trauma, pre-term birth, preeclampsia, and the necessity of surgical delivery [3,4,5]. GDM not only affects the mother’s physical health during pregnancy but also contributes to complications that can influence the delivery process and postpartum recovery. In addition to pregnancy-related complications, GDM is associated with postpartum risks such as postpartum hemorrhage, dystocia, gestational hypertension, and prenatal depression [3,6]. The hormonal and metabolic disruptions caused by GDM contribute to these adverse outcomes, potentially requiring prolonged medical attention and increasing the burden on healthcare resources.

Mothers with a history of GDM face heightened long-term risks, including the recurrence of GDM in subsequent pregnancies, metabolic syndrome, and cardiovascular disease. Furthermore, renal disease and ophthalmic conditions, such as glaucoma and diabetic retinopathy, are more prevalent in these individuals. Additionally, studies suggest an increased risk of malignancies, particularly ovarian, endometrial, and breast cancer, in women with a history of GDM [7,8]. These long-term risks highlight the need for regular follow-up and preventative measures in mothers who have experienced GDM.

GDM also poses significant risks to the fetus during pregnancy. One of the most common complications is fetal macrosomia, characterized by excessive fetal growth, which can lead to delivery-related injuries and complications for both the mother and the newborn [9].

Fetal and neonatal complications in gestational diabetes include the following: perinatal mortality, congenital malformations, macrosomia, shoulder dystocia, and birth injuries [10,11]. The intrauterine environment plays a crucial role in these outcomes, as elevated glucose concentrations in the mother lead to enhanced fetal insulin secretion. This hyperinsulinemic state contributes to increased adiposity during infancy, which predisposes the child to obesity and its associated morbidities later in life [12].

Numerous risk factors for GDM, including advanced maternal age, maternal obesity and overweight, ethnicity, prior history of GDM, and a family history of type 2 diabetes mellitus (T2DM), have been discovered by epidemiological research. However, there is still work to be done to control the long-term effects of GDM in both the mother and the child [7]. This is mainly due to the lack of adequate characterization of the molecular pathways underlying the pathogenesis of GDM [13].

Growing research indicates that the homeostatic control of glucose metabolism is significantly influenced by the intestinal gut microbiota and microbial metabolites [14]. There are several ways in which dysbiosis of the gut microbiota contributes to the development of insulin resistance and glucose intolerance in T2DM. These include imbalanced production of branched-chain amino acids and short-chain fatty acids (SCFAs) and low-grade endotoxemia due to increased gut permeability, as well as disrupted bile acid metabolism [15]. Numerous studies have identified several risk variables, such as a decline in *Coprococcus* or the presence of *Clostridium* bacteria in the microbiota, that may be associated with the development of GDM and gut dysbiosis [16,17,18,19].

The gut microbiota may be a therapeutic target for glycemic control and act as a major mediator for the therapeutic advantages of anti-diabetic medications, according to data from T2DM research [20]. T2DM and GDM are strongly associated, according to epidemiological statistics [21]. Pathophysiological, GDM and T2DM are similar in that they both have insulin resistance concurrent with impaired insulin actions and malfunction of the pancreatic β-cell [13]. However, little is known about how the gut microbiota and GDM interact.

The fetoplacental unit, which is the primary driver of this adaptive response to hormonal and metabolic alterations, is linked to pregnancy. Insulin signaling adapts to maintain normoglycemia in early pregnancy and ensures a sufficient supply of nutrients to the fetus, including increased insulin secretion from pancreatic β-cells and a blunted response to insulin-stimulated glucose uptake in peripheral tissues, especially skeletal muscle. Insulin signaling functions as a master regulator to coordinate systemic glucose homeostasis [13].

One known cause of hyperglycemia during pregnancy is insulin resistance. A growing body of research indicates that modifications to the gut microbiota are important in mediating the emergence of insulin resistance in the context of obesity and T2DM [15]. While several observational studies using the gut microbiome alone have demonstrated that GDM patients clearly have altered microbiome signatures, the precise mechanisms underlying altered gut microbiome and the onset of GDM remain unclear. The integrated analyses of microbial metabolites, gut microbiome, and host phenotype may represent a promising strategy to mine these high-dimensional data and yield mechanistic insight into the development of GDM and other multifactorial metabolic diseases, since different circulating metabolites act as intermediaries between gut microbiome and host biology [15,22,23,24].

## 2. Methodology

To conduct this narrative review, a systematic search was performed using PubMed, Scopus, and Web of Science to identify relevant studies on the correlation between gut microbiota composition, MNT, and insulin resistance in pregnancy. The search strategy included the following keywords: “gut microbiota”, “pregnancy”, “gestational diabetes mellitus”, “insulin resistance”, “medical nutrition therapy”, “probiotics”, and “dysbiosis”. The inclusion criteria were as follows: full-text articles published in English between 2000 and 2024, studies focusing on human subjects with some supporting evidence from animal models, original research articles, randomized controlled trials, systematic reviews, and meta-analyses. Exclusion criteria included the following: preprints, conference abstracts, non-peer-reviewed studies, and articles focusing solely on non-pregnant populations without direct relevance to gestational insulin resistance. Following the PRISMA guidelines, an initial screening of titles and abstracts was conducted, followed by a full-text evaluation of eligible studies. Studies were selected based on relevance, methodological quality, and significance in explaining the role of gut microbiota in pregnancy-related insulin resistance. This methodology ensures that the review provides a comprehensive, evidence-based perspective on the relationship between gut microbiota, MNT, and insulin resistance during pregnancy.

## 3. Intestinal Microbiota and Host Metabolic Interactions During Pregnancy

In recent years, numerous studies have emphasized that the microbiota is shaped by factors such as environment, diet, weight, hormones, and more, all of which impact metabolism, immunity, and overall health [25]. During pregnancy, dysbiosis can arise due to both physiological and pathological conditions, including weight gain, major metabolic shifts, and changes in the immune system [25,26]. The phyla *Bacteroides* and *Firmicutes*, which are essential for energy harvesting and metabolism, predominate in the early stages of pregnancy, and the microbiota is more diverse and stable [27,28]. Reduced microbial diversity, increased levels of pro-inflammatory bacteria (such as *Escherichia coli*), which are believed to prime the body for labor and delivery, and an increase in *Proteobacteria* and *Actinobacteria*, which support lipid metabolism and energy storage, occur later in pregnancy [29]. The microbiota’s activity and makeup are significantly influenced by diet. Pregnancy-related changes in dietary patterns, fiber consumption, and macronutrient intake might result in significant microbiome alterations [30]. High-carb or high-sugar diets during pregnancy can decrease the number of good butyrate-producing bacteria (like *Faecalibacterium*), which support the integrity of the gut barrier and lower systemic inflammation, and increase the number of *Proteobacteria*, which are linked to inflammation and insulin resistance [31]. In addition to promoting endotoxemia (the release of bacterial toxins into the circulation) as a result of a compromised intestinal barrier, high-fat diets are associated with gut dysbiosis through an increase in the *Firmicutes/Bacteroidetes* ratio, which is linked to obesity and metabolic syndrome [32]. Pregnant women who consume a plant-based, high-fiber diet may encourage the growth of beneficial SCFA producers like *Akkermansia muciniphila* and *Roseburia* [33,34]. SCFAs are essential for controlling maternal metabolism, lowering inflammation, and promoting the growth of the fetus [35].

During the prenatal and pregnancy periods, various physiological changes in the endocrine, cardiovascular, respiratory, renal, and digestive systems occur to support normal fetal development. For instance, levels of human chorionic gonadotropin (hCG) rise, stimulating the corpus luteum to produce progesterone and estrogen and playing a role in vessel formation and placental development. Placental hormones, such as lactogen, can increase insulin production and insulin resistance, making it essential for pregnant women to manage carbohydrate intake to prevent elevated blood glucose levels [25,36,37,38,39].

Elevated levels of progesterone and estrogen also promote the relaxation of vascular smooth muscle, resulting in circulatory and cardiovascular changes, such as temporary increases in blood pressure. Additionally, the activation of the renin–angiotensin–aldosterone system during pregnancy enhances sodium and water retention, which can dilute red blood cell counts, leading to anemia and an increased demand for iron [40,41,42].

Higher levels of progesterone and increased intra-abdominal pressure from uterine growth contribute to respiratory changes during pregnancy that facilitate gas exchange across the placenta. These changes include elevated tidal volume, ventilation, and respiratory rate, accompanied by decreases in expiratory reserve volume, total lung capacity, and functional residual [25,43,44].

During pregnancy, changes in the renal system, such as increased renal blood flow and a secondary rise in glomerular filtration rate, can result in conditions like proteinuria (due to enhanced glomerular filtration, increased protein transport, and reduced reabsorption of filtered protein), glucosuria (from decreased glucose reabsorption in the proximal tubule), and hyponatremia (linked to elevated hCG levels) [45,46,47,48].

These physiological modifications can result in inflammatory and immune changes that affect gut function and bacterial composition. Elevated levels of estrogen and progesterone can influence bacterial metabolism, growth, and virulence. For instance, pregnant women face a heightened risk of infections from *Listeria monocytogenes*, which can lead to adverse outcomes such as preterm delivery. Some researchers have drawn comparisons between the metabolic changes that typically occur during pregnancy and those associated with metabolic syndrome outside of pregnancy [49,50].

The gut microbiota in humans undergoes significant changes throughout life, with the adult microbiome being primarily composed of the phyla *Actinobacteria*, *Bacillota*, a phylum of mostly Gram-positive bacteria previously known as *Firmicutes*, and *Bacteroidetes* [25]. At the beginning of pregnancy, the gut microbiota resembles that of a non-pregnant woman. During pregnancy, elevated progesterone levels influence the maternal microbiome, often increasing *Bifidobacterium*, *Proteobacteria*, and *Actinobacteria*. In the third trimester, alpha diversity tends to decrease, while beta diversity and the presence of opportunistic pathogens increase. Although these changes are generally considered temporary and insignificant, alterations in the gut microbiome can have a direct impact on maternal metabolism [25,29,51,52].

During pregnancy, gut microbiota can also be affected by individual factors such as dental health (e.g., periodontal disease), inflammatory bowel disease (IBD), obesity, gestational hypertension, preeclampsia or maternal stress, which may increase the risk of spontaneous premature delivery. Studies have shown differences in beta diversity between the gut microbiota of mothers who delivered preterm and those with normal deliveries, with preterm deliveries being associated with lower microbial diversity, particularly in *Bifidobacterium* and *Streptococcus* populations [53,54].

Increased inflammation during implantation and labor—characterized by a rise in gut bacteria associated with inflammatory states in nearly 70% of women—along with decreased inflammation during mid-pregnancy, can affect the gut microbiota by raising levels of pro-inflammatory cytokines (such as IFN-γ, IL-2, IL-6, and TNF-α) and white blood cells [40]. While this is generally regarded as a physiological state, conditions such as maternal obesity, GDM, or increased intestinal permeability can result in excessive inflammation that may lead to secondary vascular dysfunction in placental tissue, fetal growth restriction, and preeclampsia. Several mechanisms contribute to the immunological changes in the gut microbiota during pregnancy, including the role of lipopolysaccharides (LPSs) from Gram-negative bacteria. These substances can elevate the production of inflammatory mediators and prostaglandins through ascending inoculation via the vagina or through the hematogenous spread of microbes from a leaky gut to the placenta or uterus [49,55].

A study by DiGiulio et al. examined 204 pregnant women with preterm prelabour rupture of membranes and found that microbial invasion of the amniotic cavity can influence the gestational state. PCR analysis identified several taxa linked to the gastrointestinal tract, including *Coprobacillus* sp. The results indicated that a positive PCR test could predict lower mean birth weights and higher rates of respiratory distress syndrome and necrotizing enterocolitis (*p* < 0.05). This study underscores the role of gut microbiota in intrauterine infections [56].

The gut microbiota during pregnancy is significantly influenced by both diet and genetics. For instance, a Western diet can lead to weight gain and dysbiosis, resulting in negative maternal outcomes. In contrast, diets rich in low-fat protein, organic proteins, fruits and vegetables, unsaturated fatty acids, whole grains, and specific strains of probiotics can promote gut microbiota health, enhance intestinal integrity, and reduce excessive systemic inflammation. Additionally, SCFAs strengthen the connections between intestinal epithelial cells, showing a negative correlation with body mass index while positively impacting metabolic changes during pregnancy, including maternal weight gain, glucose metabolism, and levels of various metabolic hormones [49,57].

Gomez-Arango et al. analyzed the fecal microbiota of 29 overweight and 41 obese pregnant women and found significant differences in metabolic hormone levels and microbiome profiles between the two groups. The researchers discovered that changes in certain metabolic hormone levels were correlated with variations in the abundance of specific microbes. For instance, *Ruminococcaceae* (ρ = 0.41, *p* < 0.0001) and *Lachnospiraceae* (ρ = 0.45, *p* < 0.0001) showed strong correlations with adipokine levels. In contrast, *Collinsella* (ρ = 0.36, *p* = 0.003) was associated with insulin, and *Coprococcus* (ρ = 0.47, *p* < 0.0001) correlated with gastrointestinal polypeptide levels. Furthermore, the study indicated that butyrate-producing bacteria could reduce levels of plasminogen activator inhibitor-1 and systolic and diastolic blood pressure. Overall, this research underscores the significant impact of gut microbiome composition on metabolic processes during pregnancy [49,58].

The differences in gut microbiota composition between healthy pregnancies and those complicated by dysbiosis-related issues such as GDM, preterm delivery, or preeclampsia are listed in Table 1.

During the prenatal period, the gut microbiome and the brain–gut axis play crucial roles in influencing maternal and child health. Negative disruptions in this balance are associated with a higher risk of chronic intestinal disorders, weight and growth deficiencies, and even neuropsychiatric disorders. Therefore, a high-quality diet and maintaining gut eubiosis are strongly recommended. Additionally, factors such as stress, infections, and antibiotic use can promote dysbiosis, increasing the risk of neurodevelopmental disorders; thus, it is essential to discuss dietary and lifestyle patterns with the mother. Furthermore, obesity or excessive weight gain during pregnancy can exacerbate metabolic changes and elevate the risk of GDM, fetal macrosomia, or preeclampsia [59,60,61].

In a study by Brantsaeter et al., the authors examined the association between the consumption of milk-based probiotic products (specifically *Lactobacillus rhamnosus GG* and *Lactobacillus acidophilus*) during pregnancy and the development of preeclampsia in a cohort of 33,399 primiparous women. The findings indicated that the intake of probiotic milk products was linked to improved glucose metabolism and a reduced risk of preeclampsia, particularly severe preeclampsia, with an odds ratio (OR) of 0.79, 95% CI: 0.66, 0.96). Furthermore, daily intake of probiotics was associated with an even lower risk (OR = 0.80, 95% CI: 0.66, 0.96) [62].

The hormonal, immunological, and metabolic changes during pregnancy impact the composition of the gut microbiota, which influences these changes. Dysbiosis can lead to pregnancy complications such as GDM, preterm delivery, and preeclampsia. Therefore, appropriate manipulation of the microbiota through diet, probiotics, and lifestyle adaptations is essential to support the health of both the mother and the fetus during pregnancy [25].

**Figure 1:**
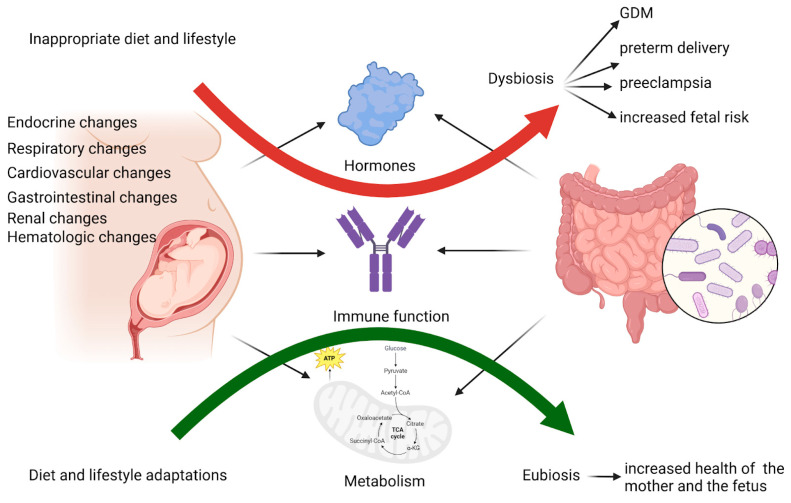
During pregnancy, various physiological changes occur in the endocrine, cardiovascular, respiratory, renal, and digestive systems. These changes lead to hormonal, immunological, and metabolic alterations that impact the gut microbiota composition. The intestinal microbiome, in turn, can influence these changes, affecting normal pregnancy progression. An inappropriate diet or lifestyle during pregnancy can disrupt the normal gut microbiota (dysbiosis), negatively influencing maternal and fetal health and increasing pregnancy risks. Conversely, appropriate manipulation of the microbiota through dietary and lifestyle adaptations promotes a eubiotic state, enhancing the health of both the mother and the fetus during pregnancy. Created with BioRender.com (accessed on 6 October 2024).

A brief overview of this chapter is depicted in Figure 1.

## 4. Changes in Gut Microbiota in Pregnancies Complicated with Insulin Resistance and GDM

During pregnancy, GDM can have short- and long-term negative effects on both the mother and the fetus. Early increases in adiposity and reduced insulin sensitivity, primarily caused by diabetogenic hormones from the placenta in the third trimester, are part of the body’s normal adaptation to support fetal growth and meet the energy demands of lactation. At the same time, physiological insulin secretion helps regulate glucose levels. Insulin resistance during pregnancy leads to immune changes, including elevated levels of circulating cytokines like TNF-α and IL-6, which are believed to contribute to obesity-related metabolic inflammation. However, when those mechanisms are insufficient, maternal hyperglycemia and GDM may occur, though the exact underlying process is not fully understood. Recent studies have suggested that the gut microbiota may play a role in insulin resistance during pregnancy, but this area requires further investigation [29,63,64].

**Figure 2:**
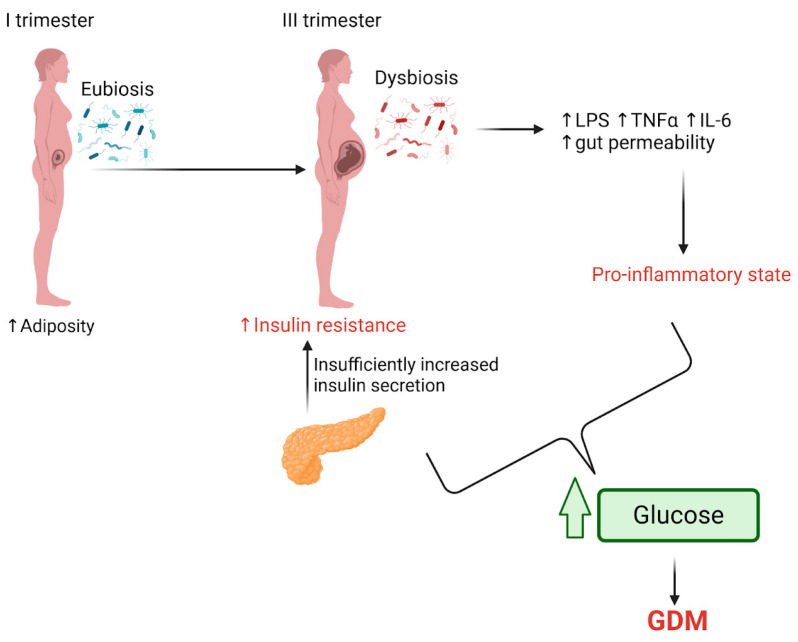
During pregnancy, early increases in adiposity and decreased insulin sensitivity typically emerge by the third trimester. Physiological increases in insulin secretion help regulate glucose levels. However, changes in gut microbiota composition from the first to the third trimester can result in dysbiosis, leading to elevated levels of LPSs from Gram-negative bacteria and immune changes, such as higher levels of TNF-α and IL-6, which contribute to a pro-inflammatory state. When insulin secretion becomes insufficient, combined with this inflammatory environment, maternal hyperglycemia and GDM may develop. Created with BioRender.com (accessed on 9 October 2024).

Figure 2 summarizes the potential mechanisms related to the development of GDM during pregnancy.

Research indicates that the gut microbiota during pregnancy can remain relatively stable or undergo significant changes to support metabolic adaptations, such as insulin resistance, and promote healthy fetal growth. These changes are marked by increased levels of *Proteobacteria* and *Actinobacteria*, a decrease in butyrate-producing bacteria, reduced bacterial richness and within-subject α diversity, and higher between-subject β diversity toward the end of pregnancy [29,65].

A study by Ferrocino et al. examined the fecal microbiota of 41 patients with GDM at 24–28 weeks and 38 weeks of gestation. The study found a significant increase in α-diversity (*p* < 0.001), with higher *Bacillota* levels and reduced *Bacteroidetes* and *Actinobacteria* levels. The study revealed a substantial decrease in *Bacteroides*, *Collinsella*, and *Rikenellaceae*, along with an increase in *Blautia*, *Butyricicoccus*, *Clostridium*, *Coprococcus*, *Dorea*, *Faecalibacterium*, *L-Ruminococcus,* and *Lachnospiraceae*. *Faecalibacterium* was inversely associated with fasting glucose (β = −1.28; 95% CI −1.71 to −0.85; *p* = 0.01), highlighting the link between inflammation and dysmetabolism during pregnancy. *Collinsella* was directly associated with insulin levels (β = 8.69; 95% CI 6 to 11.4; *p* = 0.01), while *Blautia* showed an inverse association (β = −0.42; 95% CI −0.67 to −0.17; *p* = 0.01). Both were also linked to the Homeostasis Model Assessment of Insulin Resistance (HOMA-IR) and may influence metabolism by reducing liver glycogenesis and promoting inflammation. *Sutterella* was associated with C-reactive protein levels (β = 7.57; 95% CI 5.02 to 10.1; *p* = 0.01), indicating a pro-inflammatory state. Metagenomic analysis showed enrichment in pathways related to glycolysis/gluconeogenesis, fructose and mannose metabolism, galactose metabolism, starch and sucrose metabolism, and amino acid biosynthesis, with a reduction in fatty acid metabolism, biotin metabolism, and folate biosynthesis. Additionally, a positive correlation was observed between LPS biosynthesis and *Sutterella*, *Bacteroides*, and *Phascolarctobacterium* at the end of pregnancy, which may contribute to progressive weight gain and hyperglycemia [66,67].

In a study by Koren et al., fecal bacteria from 91 pregnant women were analyzed, revealing either no difference or significant changes from the first to the third trimester. Notably, there was a marked increase in *Proteobacteria* (0.73% ± 0.08% in the first trimester to 3.2% ± 0.68% in the third trimester; *p* = 0.0004) and *Actinobacteria* (5.1% ± 0.47% to 9.3% ± 1.32%; *p* = 0.003), accompanied by reduced bacterial richness in 69.5% and 57% of women, respectively. Their findings showed higher α-diversity in the first trimester, followed by a notable expansion of β-diversity and an overall shift in microbial community composition by the third trimester. They also observed a reduction in *Faecalibacterium* levels in the third trimester. Metagenomic analysis indicated no significant differences in gene abundance or metabolic pathways between trimesters, with similar levels of *Bacteroidetes* and *Bacillota* throughout. Interestingly, the study found that GDM did not adversely affect the microbiota of the children, as the gut microbiota of children born to GDM+ mothers was similar to that of children from GDM− mothers [29].

In another study by DiGiulio et al., which analyzed vaginal, stool, saliva, and tooth/gum samples from 40 pregnant women, it was found that the taxonomic composition and diversity of the gut microbiota remained relatively stable throughout pregnancy. This suggests that the progression of pregnancy is not necessarily linked to significant changes in gut microbiota diversity and composition. However, in light of other studies showing notable shifts in gut microbiota during pregnancy, the authors emphasized the potential impact of dietary interventions between trimesters, which could influence the gut microbiota throughout pregnancy [65].

Kuang et al. analyzed fecal samples from 43 patients with GDM and 81 healthy pregnant women using whole-metagenome shotgun sequencing. Their study identified significant alterations in the gut microbiome of GDM patients, including an increased abundance of *Bacteroides* spp., *Parabacteroides distasonis*, *Klebsiella variicola*, *Megamonas*, *Phascolarctobacterium*, *Catenibacterium mitsuokai*, *Coprococcus comes*, *Enterobacteriaceae*, and *Citrobacter* spp. Conversely, they observed a significant reduction in α-diversity gene expression (*p* < 0.05), particularly in beneficial bacteria such as *Bifidobacterium* spp. (such as *B. pseudocatenulatum*, *B. animalis*, and one unclassified species), *Eubacterium* spp. (*E. siraeum*, *E. eligens*, and two unclassified *Eubacterium* species), and *Roseburia* spp. The study highlighted that the GDM-enriched bacteria observed were linked to gut dysbiosis, suggesting a direct association between GDM pathophysiology and microbiome imbalances. Additionally, GDM patients exhibited greater abundance in pathways related to membrane transport and energy metabolism, including phosphotransferase systems and LPS biosynthesis and export pathways, which correlated with glucose tolerance levels. Notably, the authors suggested that changes in microbial composition could serve as potential biomarkers for identifying individuals at risk for developing GDM [67,68].

Several shorter studies analyzing the microbiota of GDM patients have identified an increased *Bacillota/Bacteroides* ratio, along with higher levels of *Lachnospiraceae*, *Phascolarctobacterium*, and *Christensenellaceae*. *Actinobacteria* was also highlighted as a potential biomarker for GDM, accompanied by elevated levels of *Collinsella*, *Rothia*, *Actinomyces*, *Desulfovibrio*, *Leuconostoc*, *Granulicatella*, and *Mogibacterium*. Additionally, increased levels of *Bacteroides caccae*, *Bacteroides massiliensis*, and *Bacteroides thetaiotaomicron*, along with a reduction in *Bacteroides vulgatus*, *Eubacterium eligens*, *Lactobacillus rogosae*, and *Prevotella copri*, were observed in GDM patients [69,70,71].

Table 2 provides a summarized overview of the changes in microbiota composition during pregnancy.

## 5. The Role of Microbial Metabolites Resulting from an Imbalanced Microbiota

Gut microbiota dysbiosis is a condition with reduced diversity, a loss of beneficial bacteria, or an overgrowth of potential pathogenic/pathogenic microbes. This imbalance is triggered by genetic factors, health status, lifestyle choices, and environmental influences, ultimately disrupting the gut barrier and causing imbalances in the host’s immune and metabolic systems. For instance, the gut microbiota of obese individuals is typically marked by a decrease in *Bacteroidetes* and an increase in *Bacillota*, along with reduced microbial diversity and richness. This reduction is negatively correlated with the severity of metabolic markers, contributing to increased adiposity, insulin resistance, and dyslipidemia [72,73,74,75].

The interaction between gut microbiota and the host involves the production of metabolites, either directly by bacteria or through the transformation of dietary or host-derived substrates. These metabolites serve as intermediates or end-products of microbial metabolism. Microbiota-derived metabolites include bile acids (BAs), SCFAs, branched-chain amino acids (BCAAs), trimethylamine N-oxide (TMAO), tryptophan and indole derivatives, and imidazole propionate. In recent years, various metagenomics, metaproteomics, and metabolomics approaches have been developed to explore the functions of these metabolites, their pathways, and their roles in metabolic diseases more thoroughly. Many fecal and serum metabolites have been linked to metabolic disorders, and the associations between gut microbiota imbalances and metabolic disturbances have also been studied [67,75,76,77].

1. BAs are small molecules synthesized from cholesterol in hepatocytes, chenodeoxycholic acid and cholic acid (primary BAs). These are conjugated with glycine and taurine to facilitate lipid and vitamin digestion and absorption. Gut microbiota deconjugate these into secondary BAs. In patients with obesity and metabolic syndrome, BA metabolism is disrupted, particularly affecting primary BA metabolism, which contributes to hepatic steatosis and altered glucose and lipid metabolism. BAs play a crucial role in metabolic regulation by influencing serum triglyceride synthesis through the FGF19/FGF15 pathways and interacting with nuclear receptors such as through Farnesoid-X receptor (FXR) and Takeda G-protein-coupled receptor 5. Activation of these pathways increases hepatic glycogen synthesis, insulin sensitivity, pancreatic insulin secretion, energy expenditure (in the liver, brown adipose tissue, and muscles), and thermogenesis, resulting in weight loss and increased satiety in the brain. Gut microbiota dysbiosis impairs ileal absorption of BAs, normally mediated by the apical sodium-dependent bile acid transporter. This impairment reduces FXR and FGF19 expression, leading to an imbalance in BAs, particularly an increase in colonic primary conjugated Bas, which have pro-inflammatory effects on intestinal epithelial cells. These effects weaken intestinal barrier function and increase permeability through the phosphorylation occludin in intestinal Caco-2 cells. Research has shown that a high intake of animal fats increases taurocholic acid levels, stimulating the growth of sulfite-reducing bacteria like *Bilophila wadsworthia*, which raises susceptibility to colitis, exacerbates liver steatosis, impairs intestinal barrier function, and disrupts glucose metabolism. Other studies have demonstrated that secondary BA can regulate metabolic homeostasis in mice. Antibiotic supplementation, which reduces secondary BA-producing bacteria, has been found to lower hepatic concentrations of deoxycholic acid and lithocholic acid and decrease serum triglyceride levels [75,78,79,80,81].
2. SCFAs (butyrate, propionate, and acetate) are end-products of microbial fermentation with numerous physiological roles, mainly mediated through specific G protein-coupled receptors and epigenetic mechanisms. These roles include maintaining intestinal mucosal integrity, enhancing glucose and lipid metabolism, regulating energy expenditure, and modulating immune responses and inflammation. Research has shown that SCFA-producing bacteria and SCFAs are reduced in the fecal samples of obese or diabetic patients with gut dysbiosis. Supplementation with SCFAs (inulin-propionate ester, acetate, or propionate) has increased energy expenditure, improved glucose tolerance and metabolic homeostasis, and enhanced the production of GLP-1 and PYY, leading to reduced weight gain. Interestingly, some studies have shown that maternal gut microbiota, through the production of SCFAs, can activate embryonic GPR41 and GPR43 receptors, influencing the prenatal development of the neural, enteroendocrine, and pancreatic systems in offspring. This process helps maintain postnatal energy homeostasis and may prevent the growth of metabolic disorders [75,82,83,84,85,86].
3. BCAAs (valine, isoleucine, and leucine) are essential amino acids that plants, fungi, and bacteria synthesize, especially the gut microbiota. They are key in regulating protein synthesis, glucose and lipid metabolism, insulin resistance, hepatocyte proliferation, immunity, and thermogenesis in brown adipose tissue. Some studies have shown that increased calorie consumption, which can lead to gut dysbiosis, raises systemic BCAA levels and is associated with obesity and diabetes by promoting insulin resistance. Insulin resistance has been linked to elevated levels of *Prevotella copri* and *Bacteroides vulgatus* (which produce BCAAs) and reduced levels of *Butyrivibrio crossotus* and *Eubacterium siraeum* (which can utilize BCAAs) [23,75,87,88,89].
4. The gut microbiota produces TMAO through dietary choline and L-carnitine metabolism, leading to trimethylamine formation. This compound is absorbed and transported to the liver, where it is converted into TMAO by hepatic flavin monooxygenase 3. Studies have shown that elevated TMAO levels from dietary sources are directly involved in the development of metabolic diseases such as diabetes and obesity, as well as increasing the risk of cardiovascular disease and kidney failure. Research has also demonstrated that in antibiotic-treated mice with secondary dysbiosis, dietary supplementation with TMAO increases the risk of atherosclerosis [90,91,92].
5. Tryptophan is an essential aromatic amino acid obtained from the diet, involved in protein synthesis and metabolite production through three main pathways: the kynurenine pathway, the serotonin pathway, and a gut microbiota-mediated pathway that converts tryptophan into indole and its derivatives. Studies have shown that metabolic disorders and gut dysbiosis reduce the microbiota’s ability to metabolize tryptophan, leading to decreased production of GLP-1 and IL-22, increased intestinal permeability, and LPS translocation, which contribute to inflammation, insulin resistance, obesity, and liver steatosis. Other studies have found that *Lactobacillus reuteri* administration can produce aryl hydrocarbon receptor ligands that help reverse metabolic dysfunction, while indole supplementation in mice can prevent LPS-induced disruptions in cholesterol metabolism and reduce liver inflammation [75,93,94,95].
6. Imidazole propionate is a metabolite produced from the gut microbiota’s metabolism of histidine, which has been linked to insulin resistance and T2DM by disrupting the insulin signaling pathway through activation of the mammalian target of rapamycin complex 1 (mTORC1) in the liver. Some studies have also identified a connection between imidazole propionate and low-grade inflammation in individuals with an unhealthy diet and secondary dysbiosis [75,96].

Table 3 summarizes the primary gut microbiota metabolites and their metabolic effects associated with dysbiosis.

It is crucial to note that dysbiosis, which alters gut microbial metabolites, can increase ROS production. This rise in ROS can disrupt gut barrier integrity, activate the immune system, and interfere with metabolic pathways, resulting in inflammation. Additionally, ROS can exacerbate gut dysbiosis by affecting species diversity and composition [97,98,99].

Recent studies have shown promising advancements in analyzing the composition and metabolites of the gut microbiome, suggesting the potential for using these metabolites as future biomarkers for the early detection of metabolic diseases. Additionally, they may serve as targets for the development of novel therapies aimed at treating these conditions [75,76].

## 6. Diet–Microbiota Interactions in Normal and Complicated Pregnancies

When a woman has a prior medical condition or develops a new one while pregnant, it often puts both the mother and the infant at risk. The treatment of common pregnancy-related issues typically requires medical nutrition therapy (MNT). In most cases, interdisciplinary and/or multi-agency teams of healthcare providers are necessary, and referring patients to other specialists may be essential in extreme cases.

According to the most recent statistics provided by the International Diabetes Federation (IDF) in 2019, a significant number of pregnant women had glucose intolerance, making up 16% of all live births. GDM was the underlying cause of this condition in 84% of these cases, which translates to 1 out of every 6 pregnancies [100].

According to the American Diabetes Association (ADA), a diabetic patient who has not previously been diagnosed with diabetes is more likely to be diagnosed with GDM during the second or third trimester of pregnancy. Both obesity and GDM pose significant risks to public health, and when they occur during pregnancy, they are linked to a high likelihood of negative consequences [101].

While Dr. J.P. Hoet did record symptoms of prenatal hyperglycemia in 1954, it was not until the last twenty years that medical treatment guidelines and diagnostic criteria for genetically determined diabetes were established. In a major study including 23,316 pregnant women, researchers looked at hyperglycemia and adverse pregnancy outcomes (HAPO Study Cooperative Research Group) [3,102]. The International Association of Diabetes and Pregnancy Study Groups Consensus Panel revised the diagnostic criteria for GDM in 2010 based on the results of this research [103].

Pregnancy presents a window of opportunity to establish a healthy lifestyle that will ideally avoid the intergenerational transfer of obesity, metabolic syndrome, and other associated health issues. Every family should make the most of this special time. Pregnancy is a period that is unlike any other. There is a greater level of dedication among families than ever before to adopting new behaviors and selecting healthier choices. Now is the time for the medical community to take action to combat the epidemics of obesity and diabetes by putting into place effective preventative programs.

Due to the high incidence of GDM, all pregnant women must undergo testing for the illness between the 24th and 28th week of their pregnancies [104].

There is a correlation between being obese and having GDM, and both raise the risk of problems during pregnancy. The presence of these illnesses is also associated with significant public health problems. Several adverse pregnancy outcomes, such as preeclampsia, cesarean section, early delivery, and perinatal death, have been associated with maternal obesity and GDM [105,106,107,108].

Children of mothers who have GDM have an increased risk of obesity and other metabolic abnormalities, as well as an increased risk of developing T2DM after giving birth [109].

Researchers estimated that women with a history of GDM were approximately twenty percent more likely to develop T2DM by the ninth year after giving birth. According to epidemiological studies, thirty-four to eighty-four percent of pregnant women who had a history of GDM experienced a recurrence of the condition in subsequent pregnancies [110]. Additionally, twenty to forty percent of these women developed metabolic syndrome within two to twenty years, and twenty to forty percent developed obesity between five and sixteen years [6,111,112,113].

Changes in lifestyle, such as alterations to one’s diet and greater physical exercise, were the primary focus of prevention campaigns. Many people choose to make dietary adjustments as their main therapy choice. A unifying objective across nutritional treatments for prenatal hyperglycemia is to improve blood glucose management and health outcomes for breastfeeding women and their babies. Although MNT is widely acknowledged to play a role in the management of GDM, there is no consensus regarding the appropriate dietary nutrient advice for maintaining normal glucose levels in pregnant women [114]. Furthermore, the nutritional modifications necessary to prevent hyperglycemia in pregnancy vary depending on the potential risk factors.

Pregnancy causes several changes in the gut microbiota, some of which may affect the mother’s and the baby’s metabolism in the future [115]. Mothers’ hyperglycemia and obesity are associated with certain gut microbial characteristics [116]. Several medications and therapies, such as antibiotics and probiotic and prebiotic supplement non-adherence, might change metabolic function during pregnancy [117,118]. This is because these medicines impact gut microbiota, affecting overall health.

Changes to their diet may quickly and dramatically alter a person’s gut flora [119]. There may be a mediating role for microbiome profiles in the relationship between prenatal nutrition and metabolic health, as studies show that the effect of food on metabolic responses differs across individuals. These findings reveal that a diet is not always beneficial for all people or situations and that a personalized approach to human nutrition may improve outcomes. Optimizing methods for preventing and controlling metabolic illnesses during pregnancy, such as GDM and maternal obesity, depends critically on understanding how specific dietary alterations affect the makeup and function of gut microbiota [120].

During an otherwise healthy and normal pregnancy, a greater carbohydrate diet was shown to be connected with an increased risk of *Roseburia*. On the other hand, it was discovered that the intake of carbohydrates was positively correlated with the presence of *Bacteroides*, *Proteobacteria,* and *Bacillota* [121]. Barret et al. discovered that vegans demonstrated a more robust *Roseburia* than those who consume meat [122]. This was the conclusion reached by the researchers. To put this into perspective, the total quantity of carbohydrates and fiber ingested by the omnivorous and vegetarian groups was the same. It has been discovered that a lower intake of total carbohydrates is associated with a higher relative abundance of *Lachnospira* [121].

The relative abundance of *Bacillota*, *Romboutsia*, and *Proteobacteria* was lower in the microbiota of those who consumed a lot of fat [121,123].

Under normal and healthy pregnant conditions, a higher consumption of carbohydrates was shown to have a positive association with *Proteobacteria*, *Bacteroides*, and *Bacillota*, whereas it was found to have a negative association with *Roseburia* [121].

Nevertheless, there is no discernible difference between the omnivore and vegetarian groups in terms of the overall amount of carbs or fiber that they consumed [122]. There was a correlation between increasing the relative abundance of *Lachnospira* and reducing the amount of total carbs consumed [121].

A negative association was found between the consumption of total protein, particularly animal protein, and the presence of *Actinobacteria* and *Proteobacter* [121,123].

Vitamin D consumption was shown to have a favorable correlation with the presence of *Proteobacteria*. On the other hand, a higher intake of vitamin E by pregnant women who are healthy results in a decrease in the richness of this bacterial population [123].

On the other hand, it was shown that vegetarian women who eat a diet that mostly constituted carbs and contained a significant amount of fiber had a low abundance of *Collinsella* [122].

Short preterm birth (SPTB) was linked to a decline in the taxonomic class of *Betaproteobacteria* and a decrease in the α-diversity of the gut microbiota, according to a prospective study by Gershuni et al. that included 301 primiparous women who were enrolled in 20–26 weeks of gestation. The primary clinical result of spontaneous preterm versus term birth was prospectively observed after a cross-sectional assessment of food, fecal microbiota, and fecal and plasma metabolome at the second term of gestation. In pregnant women who experienced SPTB, there were noticeable increases in the excretion of lipid and steroid hormone metabolites (DHA, EPA, DHEA-S, and allopregnanolone) in the fecal metabolome. These increases were linked to higher dietary fat intake, particularly SFAs, PUFAs, and trans fatty acids [124].

Precursor fatty acid and plasma oxylipin levels during pregnancy have been proposed as a possible predictor of SPTB [125]. The current study found that women with SPTB had a higher ratio of 14-HDoHE to 17-HDoHE; however, it is unclear what this difference means during pregnancy. Crucially, based on food recall data, these analytical traits aligned with the modest dietary changes between cases and controls [124].

A high-fat, low-fiber diet has been associated with an increased risk of SPTB, while a lower-fat, higher-fiber diet has been associated with a decreased risk, according to large multinational cohort data [126,127,128].

Returning to the importance of MNT in GDM, there are several ways in which this treatment can improve a pregnant woman’s physical health. These include preventing maternal or infant problems, lowering insulin reliance, preventing postpartum diabetes, preventing child macrosomia, preventing the development of adult metabolic syndrome, obesity, or glucose intolerance, or providing glycemic control by dietary modification. MNT is customized according to blood glucose trends, activity levels, and pre-pregnancy BMI [129,130]. This guarantees that it meets the energy requirements of the mother as well as glycemic management. Emphasis is placed on meal timing and frequency (three small meals and two to four snacks) to avoid nocturnal hypoglycemia or fasting hyperglycemia. The distribution of calories consists of 40–50% carbs, 20–25% proteins, and 25–30% healthy fats [131].

High-fiber diets, a cornerstone of MNT, promote the growth of beneficial gut bacteria (e.g., *Bifidobacterium* and *Lactobacillus*). These bacteria ferment fiber to produce SCFAs, such as butyrate, which enhance insulin sensitivity and reduce inflammation [132].

MNT often incorporates probiotics (live beneficial bacteria) and prebiotics (substances that feed beneficial bacteria). Probiotic supplementation has been shown to improve glucose metabolism and modulate gut microbiota composition, which is advantageous for insulin resistance [133].

Limiting refined carbohydrates and sugars can prevent the overgrowth of pathogenic bacteria associated with metabolic dysfunction [134]. According to research, well-structured MNT can improve postprandial glycemic responses, lower fasting glucose levels, and lessen the requirement for pharmacologic treatments (such as insulin or oral hypoglycemic medications) [135,136]. Limiting simple sugars, using low-glycemic index carbs to postpone the absorption of glucose, and distributing carbohydrates evenly across meals and snacks are important tactics in this regard [137,138].

In many cases of GDM, MNT has been demonstrated to lessen or completely eliminate the need for exogenous insulin. With dietary changes alone, 70–85% of women with GDM can attain appropriate glucose control [129,139,140]. When MNT is unable to sustain desired blood glucose levels, insulin therapy is usually saved for certain situations [141].

Omega-3 fatty acids, found in certain fish and seeds, and antioxidants from fruits and vegetables can reduce gut inflammation, a key driver of insulin resistance [142].

When possible, MNT should be individualized based on gut microbiota analysis. Regular blood glucose monitoring and dietary adjustments ensure optimal outcomes for maternal and fetal health.

Regarding the short and long-term outcomes of MNT for the mother and fetus or the future young adult, there are several aspects to be taken into consideration, such as the capacity of MNT in maintaining maternal blood glucose levels, lowering the risk of hypertensive disorders, preeclampsia, and cesarean deliveries [130,138,143,144]. By preventing prenatal overexposure to glucose, controlled glucose levels lower the incidence of shoulder dystocia, macrosomia (birth weight greater than 4000 g), neonatal hypoglycemia, and long-term obesity [145,146]. In comparison to uncontrolled GDM, MNT can lower macrosomia rates by up to 50% [147].

In conclusion, MNT is a proven, non-invasive, and successful treatment that targets the primary metabolic abnormalities in GDM. It is essential for maintaining a balance between the health of the mother and the fetus, reducing both immediate and long-term dangers, and equipping women with enduring eating habits. MNT and gut microbiota are interconnected in managing insulin resistance during pregnancy. Optimizing diet to enhance gut health can make MNT crucial in improving metabolic outcomes in pregnant women.

## 7. Conclusions and Future Perspectives

The intricate interplay between the gut microbiota, MNT, and metabolic health during pregnancy underscores its pivotal role in shaping maternal and fetal outcomes. Throughout pregnancy, physiological and hormonal changes drive significant shifts in the gut microbiota composition, with increased levels of *Bifidobacterium*, *Proteobacteria*, and *Actinobacteria*, alongside reduced microbial diversity in late gestation. While physiologically adaptive, these changes may be exacerbated by factors such as poor dietary choices, obesity, and GDM, leading to gut dysbiosis, increased inflammation, and metabolic imbalances. The evidence reviewed highlights how dysbiosis, marked by reduced beneficial bacteria such as *Faecalibacterium* and *Akkermansia muciniphila*, and an overgrowth of pro-inflammatory species like *Collinsella* and *Proteobacteria*, contributes to insulin resistance, hyperglycemia, and adverse pregnancy outcomes such as preeclampsia, macrosomia, and preterm delivery.

MNT emerges as a cornerstone in addressing these challenges by directly targeting both glycemic control and microbiota composition. High-fiber diets, rich in prebiotics, promote the growth of SCFA-producing bacteria, such as *Roseburia* and *Lactobacillus*, which enhance gut barrier integrity, reduce systemic inflammation, and improve insulin sensitivity. Probiotic supplementation, particularly strains like *Bifidobacterium* and *Lactobacillus rhamnosus*, has demonstrated promise in modulating gut microbiota composition, improving glucose metabolism, and reducing the risk of GDM-related complications. Furthermore, individualized dietary interventions, such as limiting high-fat and high-sugar diets while emphasizing low-glycemic-index carbohydrates, have stabilized maternal glucose levels, reduced excessive weight gain, and mitigated inflammatory pathways.

The long-term implications of maternal gut health and MNT extend beyond pregnancy, influencing the metabolic health of offspring. Studies indicate that maintaining a eubiotic gut environment through dietary and lifestyle modifications during pregnancy can decrease the risk of childhood obesity, metabolic syndrome, and T2DM in the offspring. These findings emphasize the intergenerational benefits of gut microbiota optimization and MNT.

Future research should aim to identify specific gut microbial signatures as biomarkers for early detection of insulin resistance and GDM, enabling timely interventions. The integration of advanced multi-omics approaches, including metagenomics, metabolomics, and transcriptomics, holds the potential to unravel the complex interactions between gut microbiota, microbial metabolites, and host metabolism. Such insights could pave the way for precision medicine strategies, leveraging tailored probiotics, prebiotics, and personalized MNT to optimize maternal and fetal health outcomes.

In conclusion, the synergy between gut microbiota and MNT represents a promising avenue for transforming the management of insulin resistance and metabolic health in pregnancy. By prioritizing gut health through evidence-based dietary interventions, we can improve immediate pregnancy outcomes and lay the foundation for long-term health benefits for both mother and child.

Customizing MNT according to each person’s unique gut microbiota composition can completely transform the treatment of GDM. Future MNT procedures may use probiotic/prebiotic supplements or tailored dietary interventions to modify gut composition, enhance insulin sensitivity, and lower the risk of GDM by identifying particular bacterial strains associated with insulin resistance [148].

Future studies may aim to find particular gut microbial profiles as early indicators of insulin resistance during pregnancy. By enabling prompt dietary or pharmaceutical changes, early diagnosis via stool analysis or metabolite profile may stop the development of insulin resistance and its related problems.

Improving insulin sensitivity and controlling glucose metabolism may be possible by developing specific probiotics or prebiotics that boost advantageous bacterial strains like *Bifidobacterium* or *Akkermansia muciniphila.* Clinical trials are required to determine the best strains, dosages, and times to administer medication during pregnancy.

Future research should examine the effects of maternal insulin resistance and gut microbiota on the long-term metabolic health of offspring. Understanding these intergenerational impacts could lower the likelihood of childhood obesity and metabolic diseases and inform dietary recommendations for pregnant women.

During the fermentation of fiber, gut bacteria produce SCFAs, which are essential for insulin sensitivity and glucose metabolism [149]. Future studies could examine the effects of dietary approaches that increase the formation of SCFAs on insulin resistance during pregnancy and the potential medicinal uses of these metabolites.

By integrating genomes, metabolomics, and metagenomics, we will gain an extensive understanding of how gut microbiota affects insulin resistance. These technologies can expand our knowledge of host–microbe interactions during pregnancy and identify new treatment targets.

Future research could examine the relationship between the composition of the gut microbiota and the effectiveness of drugs like metformin that are used to treat insulin resistance. Comprehending these interplays could enhance medication treatments and reduce adverse effects.

Given that the gut flora changes during pregnancy, future studies might create nutritional recommendations tailored to each trimester. This strategy might guarantee that dietary interventions align with the changing requirements of the mother’s metabolic state and microbiome.

Maternal care programs should be enhanced by incorporating microbiome research into public health strategies, especially for groups at high risk for GDM. It may become commonplace in prenatal care to provide education and resources that support gut health through food.

For the future, MNT and gut microbiota manipulation offer a potential tool for treating insulin resistance in pregnancy. Targeted probiotics, early biomarkers, and personalized medicines can revolutionize prenatal care and enhance outcomes for expectant mothers and their unborn children. 

## Figures and Tables

**Table 1:** An extensive list of gut microbiota composition during healthy and complicated pregnancies.

Condition	Gut Microbiota Composition		References
	Increased	Decreased	
Healthy Pregnancy	↑ Beta diversity *↑ Bifidobacteria* *↑ Actinobacteria* *↑ Proteobacteria* *↑ Bacillota/Bacteroides ratio* *↑ Blautia* *↑ Collinsela* *↑ Bifidobacterium*	↓ Alpha diversity ↓ *Acinetobacter* *↓ Bacteroides* *↓ Parabacteroides*	[51,52]
Preeclampsia	*↑ Fusobacterium* *↑ Veillonella* *↑ Bulleidia moorei* *↑ Clostridium perfringens*	*↓ Faecalibacterium* *↓ Akkermansia* *↓ Coproccocus catus*	[25,51]
GDM	*↑ Ruminococcaceae* *↑ Enterobacteriaceae* *↑ Prevotella* *↑ Collinsella* *↑ Parabacteroides distasonis* *↑ Bacillota/Bacteroides ratio* *↑ Lachnospiraceae* *↑ Phascolarctobacterium* *↑ Christensenellaceae*	↓ *Bifidobacterium* *↓ Faecalibacterium* *↓ Akkermansia* *↓ Bacteroides vulgatus* *↓ Eubacterium eligens* *↓ Lactobacillus rogoase* *↓ Prevotella copri*	[25,51,52]
Obesity	*↑ Bacillota* ↑ Inflammatory markers ↑ *Actinobacteria*	↓ *Bifidobacterium* ↓ Alpha diversity	[25,51]

**Table 2:** Changes in microbiota composition during pregnancy.

**Study**	Increased Species	Decreased Species	Healthy/Pathological Pregnancy
Ferrocino et al. [66]	α-diversity *Bacillota* *Blautia* *Butyricicoccus* *Clostridium* *Coprococcus* *Dorea* *Faecalibacterium* *L-Ruminococcus* *Lachnospiraceae* *Sutterella* *Phascolarctobacterium*	*Bacteroidetes* *Actinobacteria* *Bacteroides* *Collinsella* *Rikenellaceae*	Healthy pregnancy
Koren et al. [29]	α-diversity—I trimester β-diversity—III trimester *Proteobacteria* *Actinobacteria*	*Faecalibacterium*	Pathological pregnancy
DiGiulio et al. [65]	No significant changes in gut microbiota diversity and composition		Healthy pregnancy
Kuang et al. [68]	*Bacteroides* spp. *Parabacteroides distasonis* *Klebsiella variicola* *Megamonas* *Phascolarctobacterium* *Catenibacterium mitsuokai* *Coprococcus comes* *Enterobacteriaceae* *Citrobacter* spp.	*Bifidobacterium* spp. (*B. pseudocatenulatum*, *B. animalis*, one unclassified) *Eubacterium* spp. (*E. siraeum*, *E. eligens*, two unclassified *Eubacterium* species) *Roseburia* spp.	Pathological pregnancy
Cortez et al. [69]	*Bacillota/Bacteroides* ratio *Lachnospiraceae* *Phascolarctobacterium* *Christensenellaceae*	NA	Pathological pregnancy
Crusell et al. [70]	*Actinobacteria* *Collinsella* *Rothia* *Actinomyces* *Desulfovibrio* *Leuconostoc* *Granulicatella* *Mogibacterium*	NA	Pathological pregnancy
Festa et al. [71]	*Bacteroides caccae* *Bacteroides massiliensis* *Bacteroides thetaiotaomicron*	*Bacteroides vulgatus* *Eubacterium eligens* *Lactobacillus rogosae* *Prevotella copri*	Pathological pregnancy

**Table 3:** Gut microbiota metabolites and their metabolic effects associated with dysbiosis.

Metabolites	Metabolic Effects	References
BAs	hepatic steatosis alter glucose and lipid metabolism pro-inflammatory effects	[75,78,79,80,81]
SCFAs	alter intestinal mucosal integrity alter glucose and lipid metabolism pro-inflammatory effects immune effects	[69,76,77,78,79,80]
BCAAs	alter protein, glucose, and lipid metabolism increase insulin resistance immune effects	[69,81,82,83,84]
TMAO	increased risk of diabetes, obesity, cardiovascular disease, and kidney failure	[85,86,87]
Tryptophan and indole derivatives	alter protein metabolism pro-inflammatory effects increase insulin resistance liver steatosis	[75,93,94,95]
Imidazole propionate	increase insulin resistance low-grade inflammation	[75,96]

## Abbreviations

Bas	bile acids
BCAAs	branched-chain amino acids
FXR	Farnesoid-X receptor
GDM	gestational diabetes mellitus
hCG	human chorionic gonadotropin
HOMA-IR	Homeostasis Model Assessment of Insulin Resistance
LPS	lipopolysaccharide
MNT	medical nutritional therapy
SCFAs	short-chain fatty acids
T2DM	type 2 diabetes mellitus
TMAO	trimethylamine N-oxide
